# A Robust, Simple Genotyping-by-Sequencing (GBS) Approach for High Diversity Species

**DOI:** 10.1371/journal.pone.0019379

**Published:** 2011-05-04

**Authors:** Robert J. Elshire, Jeffrey C. Glaubitz, Qi Sun, Jesse A. Poland, Ken Kawamoto, Edward S. Buckler, Sharon E. Mitchell

**Affiliations:** 1 Institute for Genomic Diversity, Cornell University, Ithaca, New York, United States of America; 2 Computational Biology Service Unit, Cornell University, Ithaca, New York, United States of America; 3 Hard Winter Wheat Genetics Research Unit, United States Department of Agriculture/Agricultural Research Service, Manhattan, Kansas, United States of America; 4 Plant, Soil and Nutrition Research Unit, United States Department of Agriculture/Agricultural Research Service, Ithaca, New York, United States of America; Temasek Life Sciences Laboratory, Singapore

## Abstract

Advances in next generation technologies have driven the costs of DNA sequencing down to the point that genotyping-by-sequencing (GBS) is now feasible for high diversity, large genome species. Here, we report a procedure for constructing GBS libraries based on reducing genome complexity with restriction enzymes (REs). This approach is simple, quick, extremely specific, highly reproducible, and may reach important regions of the genome that are inaccessible to sequence capture approaches. By using methylation-sensitive REs, repetitive regions of genomes can be avoided and lower copy regions targeted with two to three fold higher efficiency. This tremendously simplifies computationally challenging alignment problems in species with high levels of genetic diversity. The GBS procedure is demonstrated with maize (IBM) and barley (Oregon Wolfe Barley) recombinant inbred populations where roughly 200,000 and 25,000 sequence tags were mapped, respectively. An advantage in species like barley that lack a complete genome sequence is that a reference map need only be developed around the restriction sites, and this can be done in the process of sample genotyping. In such cases, the consensus of the read clusters across the sequence tagged sites becomes the reference. Alternatively, for kinship analyses in the absence of a reference genome, the sequence tags can simply be treated as dominant markers. Future application of GBS to breeding, conservation, and global species and population surveys may allow plant breeders to conduct genomic selection on a novel germplasm or species without first having to develop any prior molecular tools, or conservation biologists to determine population structure without prior knowledge of the genome or diversity in the species.

## Introduction

During the last decade, extensive public resources were dedicated to genotyping humans, a species with relatively low genetic diversity (about one substitution per thousand nucleotides) [1]–[3]. Many species including maize [4], [5], *Drosophila* [6], and some bacteria [7], however, are at least 10 times more diverse than humans (more than one substitution per hundred nucleotides). Besides containing high levels of nucleotide diversity, the maize genome also exhibits frequent transposon-mediated rearrangements that produce extensive presence/absence variation that often encompasses genic regions [8]–[10]. Standard, fixed-sequence approaches like single base extension assays or microarrays require invariant primer binding sites in order to obtain consistent results. Such invariant regions are often difficult to find in maize [11]. Furthermore, the large-scale structural variation also complicates DNA sequence alignment, resulting in a maize “reference” genome that contains only 70% or less of the species-wide genome space [12].

Although abundant diversity is a challenge to assays that rely on scoring fixed positions, it is advantageous to direct sequencing approaches because sequencing efficiency for genotyping scales directly with genetic diversity. We have developed a technically simple, highly multiplexed, genotyping-by-sequencing (GBS) approach that is suitable for population studies, germplasm characterization, breeding, and trait mapping in diverse organisms. This procedure, which can be generalized to any species at a low per-sample cost, is based on high-throughput, next-generation sequencing of genomic subsets targeted by restriction enzymes (REs).

Next-generation sequencing (NGS) technologies have been recently used for whole genome sequencing and for re-sequencing projects where the genomes of several specimens are sequenced to discover large numbers of single nucleotide polymorphisms (SNPs) for exploring within-species diversity, constructing haplotype maps and performing genome-wide association studies (GWAS) [13]. Multiplex sequencing has also been accomplished by tagging randomly sheared DNA fragments from different samples with unique, short DNA sequences (barcodes) and pooling samples into a single sequencing channel [14]. This approach (random DNA shearing followed by barcode tagging) works very well for species with small genomes, including organellar and microbial DNAs, and has been used to rapidly determine the complete chloroplast genome sequences of spruce and several pine species [15] and for discovery and mapping of genomic SNPs in rice [16], [17].

Although GBS is fairly straightforward for small genomes, target enrichment or reduction of genome complexity must be employed to ensure sufficient overlap in sequence coverage for species with large genomes. Enrichment strategies including long range PCR-amplification of specific genomic regions, use of molecular inversion probes, and various DNA hybridization/sequence capture methods [18] are time-consuming, technologically challenging, and can be cost-prohibitive for assaying large numbers of samples. Reducing genome complexity with restriction enzymes (REs), however, is easy, quick, extremely specific, highly reproducible, and may reach important regions of the genome that are inaccessible to sequence capture approaches. By choosing appropriate REs, repetitive regions of genomes can be avoided and lower copy regions can be targeted with two to three fold higher efficiency [12], [19], which tremendously simplifies computationally challenging alignment problems in species with high levels of genetic diversity.

The value of sequencing restriction site-associated genomic DNA (i.e., RAD tags) for high-density SNP discovery and genotyping was first demonstrated by Baird et al. [20]. Increased efficiency and cost benefits were realized by incorporating a multiplex sequencing strategy that uses an inexpensive barcoding system. Because barcodes are included in one of the adapter sequences (i.e., they are not added to individual DNA samples by PCR), reagent costs for constructing sequencing libraries are minimized. The location of the barcode, just upstream of the RE cut-site in genomic DNA, also eliminates the need for a second Illumina sequencing (“indexing”) read. The present work describes an even more cost-effective genotyping procedure based on NGS technology (Illumina, Inc.). The barcoding strategy is similar to RAD but modulation of barcode nucleotide composition and length results in fewer sequence phasing errors. Compared to the RAD method, the procedure described here is substantially less complicated; generation of restriction fragments with appropriate adapters is more straightforward, single-well digestion of genomic DNA and adapter ligation results in reduced sample handling, there are fewer DNA purification steps and fragments are not size selected. Costs can be further reduced via shallow genome sampling coupled with imputation of missing internal SNPs in haplotype blocks. The following protocol was initially developed for maize, a genetically diverse (see above), large genome species (2.3 Gbp) [21]. We have since used this procedure for genotyping and mapping in several other species. Results for both maize and barley are reported herein.

## Methods

### DNA Samples

Samples comprised the parents and 276 recombinant inbred lines (RILs) from a high resolution maize mapping population (IBM [22]), and the parents and 43 doubled haploid (DH) barley lines from the Oregon Wolfe Barley (OWB) mapping population [23]. The 43 barley lines were selected from the larger set of 83 OWB lines to maximize recombination. High molecular weight DNAs were extracted from leaves of single plants using a standard CTAB protocol [24].

### Choosing REs and Adapter Design

Selection of REs that leave 2 to 3 bp overhangs and do not cut frequently in the major repetitive fraction of the genome is of critical importance. A suitable RE for maize and close relatives (teosintes) is *Ape*KI, a type II restriction endonuclease that recognizes a degenerate 5 bp sequence (GCWGC, where W is A or T), creates a 5′ overhang (3 bp), has relatively few recognition sites in the major classes of maize retrotransposons, and is partially methylation sensitive (will not cut if the 3′ base of the recognition sequence on both strands is 5-methylcytosine). Using an RE that leaves an overhang comprising more than one nucleotide is extremely useful in promoting efficient adapter ligation to insert DNA.

**Figure 1:**
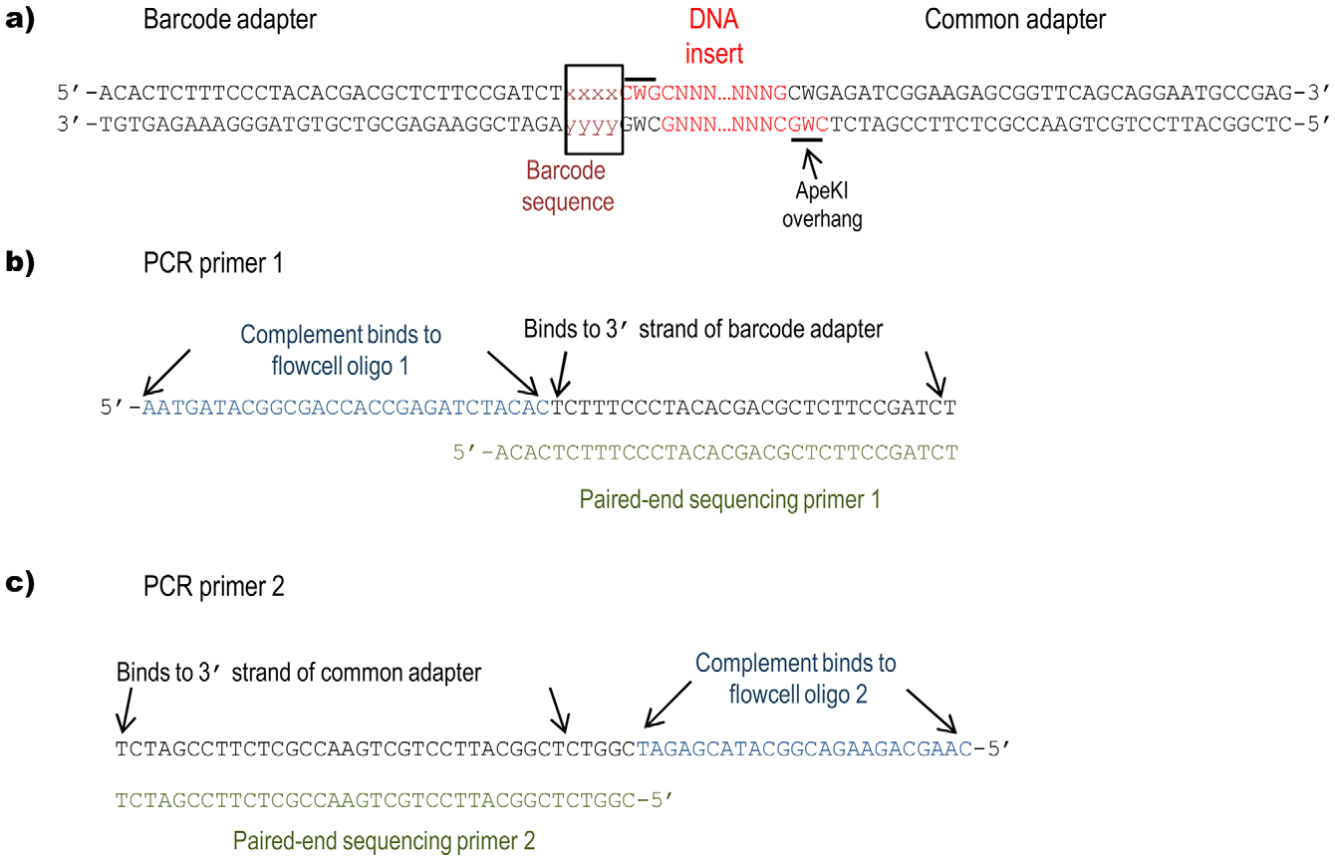
GBS adapters, PCR and sequencing primers. (a) Sequences of double-stranded barcode and common adapters. Adapters are shown ligated to *Ape*KI-cut genomic DNA. Positions of the barcode sequence and *Ape*KI overhangs are shown relative to the insert DNA; (b) Sequences of PCR primer 1 and paired end sequencing primer 1 (PE-1). Binding sites for flowcell oligonucleotide 1 and barcode adapter are indicated; (c) Sequences of PCR primer 2 and paired end sequencing primer 2 (PE-2). Binding sites for flowcell oligonucleotide 2 and common adapter are indicated.

Two different types of adapters were used in this protocol. The “barcode” adapter terminates with a 4 to 8 bp barcode on the 3′ end of its top stand and a 3 bp overhang on the 5′ end of its bottom strand that is complementary to the “sticky” end generated by *Ape*KI (CWG). The sequences of the two oligonucleotides comprising the barcode adapter are: 5′-ACACTCTTTCCCTACACGACGCTCTTCCGATCTxxxx and 5′-CWGyyyyAGATCGGAAGAGCGTCGTGTAGGGAAAGAGTGT and, where “xxxx” and “yyyy” denote the barcode and barcode complement and sequences, respectively (Figure 1). The second, or “common”, adapter has only an *Ape*KI-compatible sticky end: 5′-CWGAGATCGGAAGAGCGGTTCAGCAGGAATGCCGAG and 5′-CTCGGCATTCCTGCTGAACCGCTCTTCCGATCT (Figure 1). Adapters were designed so that the *Ape*KI recognition site did not occur in any adapter sequence and was not regenerated after ligation to genomic DNA. Adapter design also allows for either single-end or paired-end sequencing on the Illumina, Inc. (San Diego, CA) NGS platforms.

A compatible set of 96 barcode sequences that have been used for multiplex sequencing is provided as supporting information (Table S1). To minimize the possibility of misidentifying samples as a result of sequencing or adapter synthesis error, all pair-wise combinations of barcodes differed by a minimum of three mutational steps. Hence, it should be possible to correctly assign samples with single base barcode sequencing errors, or to identify particular adapters with high rates of synthesis error [25]. To avoid the potential loss of sequence quality due to phasing errors caused by reading through a non-variable restriction site prior to the twelfth base, or through an adapter position with a highly skewed base ratio [(http://www.illumina.com/Documents/products/technotes/technote_rta_theory_operations.pdf)], barcode lengths were modulated from 4 to 8 bp and care was taken to maximize the balance of the bases at each position in the overall set. For barcodes larger than 5 bases, mononucleotide runs of 3 or more, and barcodes that contained sequences of smaller barcodes were disallowed.

### Preparation of Libraries for Next-Generation Sequencing

**Figure 2:**
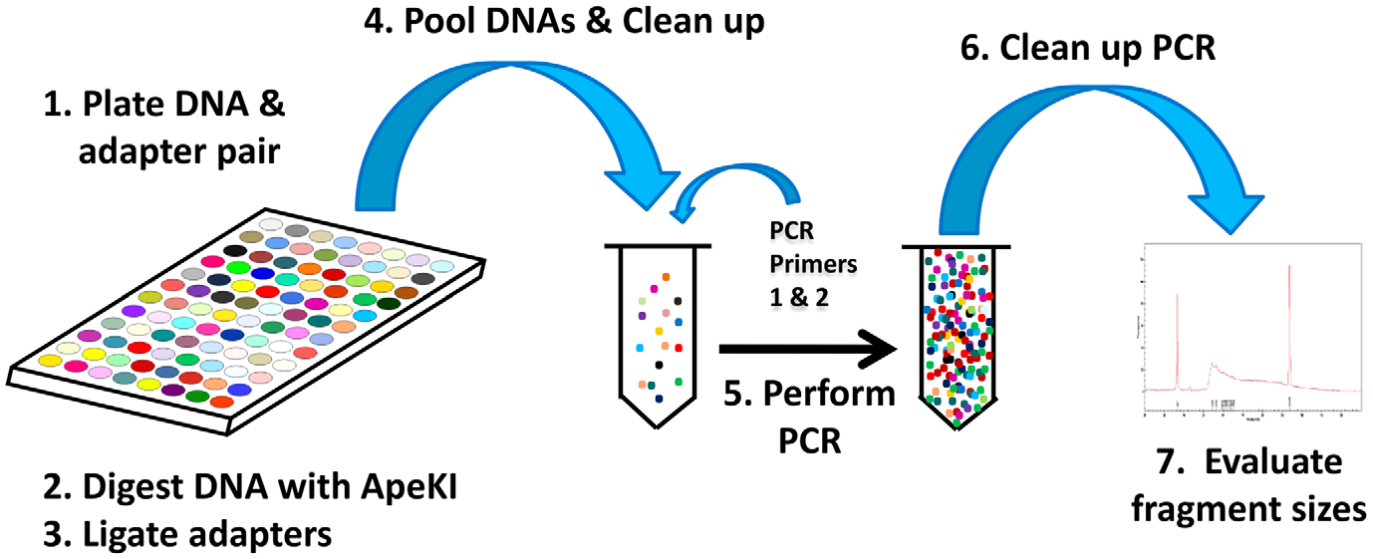
Steps in GBS library construction. Note: Up to 96 DNA samples can be processed simultaneously. (1) DNA samples, barcode, and common adapter pairs are plated and dried; (2–3) samples are then digested with *Ape*KI and adapters are ligated to the ends of genomic DNA fragments; (4) T4 ligase is inactivated by heating and an aliquot of each sample is pooled and applied to a size exclusion column to remove unreacted adapters; (5) appropriate primers with binding sites on the ligated adapters are added and PCR is performed to increase the fragment pool; (6–7) PCR products are cleaned up and fragment sizes of the resulting library are checked on a DNA analyzer(BioRad Experion® or similar instrument). Libraries without adapter dimers are retained for DNA sequencing.

A basic schematic of the protocol used for performing GBS is shown in Figure 2. Oligonucleotides comprising the top and bottom strands of each barcode adapter and a common adapter were diluted (separately) in TE (50 µM each) and annealed in a thermocycler (95°C, 2 min; ramp down to 25°C by 0.1°C/s; 25°C, 30 min; 4°C hold). Barcode and common adapters were then quantified using an intercalating dye (PicoGreen®; Invitrogen, Carlsbad, CA), diluted in water to 0.6 ng/µL (∼02 pmol/µL), mixed together in a 1∶1 ratio, and 6 µL (∼0.06 pmol each adapter) of the mix was aliquoted into a 96-well PCR plate and dried down. DNA samples (100 ng in a volume of 10 µL) were added to individual adapter-containing wells and plates were, again, dried.

Samples (DNA plus adapters) were digested for 2 h at 75°C with *Ape*KI (New England Biolabs, Ipswitch, MA) in 20 µL volumes containing 1× NEB Buffer 3 and 3.6 U *Ape*KI. Adapters were then ligated to sticky ends by adding 30 µL of a solution containing 1.66× ligase buffer with ATP and T4 ligase (640 cohesive end units) (New England Biolabs) to each well. Samples were incubated at 22°C for 1 h and heated to 65°C for 30 min to inactivate the T4 ligase. Sets of 48 or 96 digested DNA samples, each with a different barcode adapter, were combined (5 µL each) and purified using a commercial kit (QIAquick PCR Purification Kit; Qiagen, Valencia, CA) according to the manufacturer's instructions. DNA samples were eluted in a final volume of 50 µL. Restriction fragments from each library were then amplified in 50 µL volumes containing 2 µL pooled DNA fragments, 1× *Taq* Master Mix (New England Biolabs), and 25 pmol, each, of the following primers: (A) 5′-AATGATACGGCGACCACCGAGATCTACACTCTTTCCCTACACGACGCTCTTCCGATCT and (B) 5′-CAAGCAGAAGACGGCATACGAGATCGGTCTCGGCATTCCTGCTGAACCGCTCTTCCGATCT. These primers contained complementary sequences for amplifying restriction fragments with ligated adapters, binding PCR products to oligonucleotides that coat the Illumina sequencing flow cell and priming subsequent DNA sequencing reactions [26] (Figure 1).

Temperature cycling consisted of 72°C for 5 min, 98°C for 30 s followed by 18 cycles of 98°C for 30 s, 65°C for 30 s, 72°C for 30 s with a final *Taq* extension step at 72°C for 5 min. These amplified sample pools constitute a sequencing “library.” Libraries were purified as above (except that the final elution volume is 30 µL) and 1 µL was loaded onto an Experion® automated electrophoresis station (BioRad, Hercules, CA) for evaluation of fragment sizes. Libraries were considered suitable for sequencing if adapter dimers (∼128 bp in length) were minimal or absent and the majority of other DNA fragments were between 170–350 bp. If adapter dimers were present in excess of 0.5% (based on the Experion® output), libraries were constructed again using a few DNA samples and decreasing adapter amounts. Guidelines for adapting the protocol to different species including details for performing adapter titrations and are provided in Supporting Information (Text S1, Figure S1 and Figure S2).

Once the appropriate quantity of adapters was empirically determined for a particular enzyme/species combination, no further adapter titration was necessary. Single-end sequencing (86 bp reads) of one 48- or 96-plex library per flowcell channel, was performed on a Genome Analyzer II (Illumina, Inc., San Diego, CA). See Bentley et al. [26] for details of the sequencing process and chemistry.

### Filtering Raw Sequence Data

Analyses of the 86 bp sequencing reads were based upon the unfiltered qseq files, since the filtering process that produces fastq files sometimes discarded good reads that aligned perfectly to the reference genome for at least 64 bases. Starting with the qseq files from a flow cell, we first filtered for reads that (1) perfectly matched one of the barcodes and the expected four-base remnant of the *Ape*KI cut site (CWGC), (2) were not adapter/adapter dimers, and (3) contained no “Ns” in their first 72 bases. These reads were sorted into separate files according to their barcode, with the barcode removed and the remainder of the sequence trimmed to 64 bases (including the initial CWGC). If either the full *Ape*KI site (from partial digestion or chimera formation) or the first 8 bases of common adapter (from *Ape*KI fragments less than 64 bases) were detected within 64 bases, the read was truncated appropriately and then filled to 64 bases with polyA.

For maize, subsequent filtering of the reads was then done in two different ways, depending on our purpose. To generate a reference set of 64 base sequence tags to be included in a presence/absence genotype table, only reads with a minimum Q-score of 10 across the first 72 bases) and that occurred at least twice were kept. We opted to use this somewhat low-stringency minimum Q-score cutoff to maximize the number of useful sequence tags. Sequence tags containing random sequencing errors should not occur multiple times in multiple samples and should not map genetically, so they should be filtered out in subsequent steps. To this set of reference tags, the expected 64 base tags from an *in silico Ape*KI digest of the maize reference genome, B73 RefGen v1 [21], were added (with fragments shorter than 64 bases filled with polyA, as above). To fill in the observed counts in the genotype table, a second pass across the reads for each DNA sample was performed. In this second pass, 64 base reads were counted for each sample (and the count added to the genotype table) if they perfectly matched one of the reference tags, regardless of their minimum Q score. The resulting genotype table was then filtered to remove tags that occurred in 10 or fewer DNA samples; this should remove most of the sequencing errors. For barley, the absence of a reference genome prevented anchoring reads to a physical map. Sequence reads were simply filtered for unique 64 base sequence reads that were present in five or more lines and these were mapped genetically as described below.

All maize and barley sequences were submitted to the National Center for Biotechnology Information (NCBI) Short Read Archive (study SRP004282.1).

### DNA sequence alignments

The filtered sequence reads were first aligned to the maize reference genome (B73 RefGen v1) using the Burrows-Wheeler alignment tool (BWA) [27], allowing a maximum of four mismatches and one gap of up to 3 bp. The Basic Local Alignment Search Tool (BLAST) [28] was used to query reads that were not aligned by BWA, first against the maize reference genome with an e-value cutoff of 1e^−2^ and then against the National Center for Biotechnology Information (NCBI) nt database using default settings.

### Mapping

Presence/absence scores for each tag were used in a binomial test of segregation versus an independent framework map. For maize, this framework map consisted of 644 SNPs genetically mapped in the maize nested association mapping (NAM) population [29] and then genotyped in the IBM population. The binomial segregation test filtered for sequence tags that co-segregated with only one of the two parental alleles at a given SNP. For each SNP marker, the two possible parental sources of a tag were each tested in turn. A “success” was recorded when a tag co-occurred in a RIL with the SNP allele from its presumed parental source, otherwise a “failure” was recorded. The binomial sample size was the number of RILs in which the tag was present and the SNP was not missing or heterozygous. For maize, tests were only performed if the sample size was at least 10. The probability of success was defined as the proportion of the RILs that contained the SNP allele being tested. For maize, a threshold *p*-value of 0.001 was considered significant for directed tests versus the physically closest SNP, or 0.0001 for elsewhere in the genome.

For barley, mapping was conducted using flanking SNPs and a threshold of *p*<0.0001 for the binomial test. In practice, a sequence tag was mapped in barley only if it always co-occurred with one SNP allele and never the other.

In maize only, biallelic GBS markers were identified as follows. Pairs of tags that aligned to exactly the same unique position and strand in the maize reference genome (B73 RefGen v1) and that also co-segregated with the physically closest SNP (*p*<0.001) were merged into a single, biallelic marker. These markers were then re-tested for co-segregation with the physically closest SNP using Fisher's Exact Test (*p*<0.001). Biallelic GBS markers that passed the latter test were then incorporated into a high density, framework map and ordered according to their positions in the reference genome. To determine how many of the remaining presence/absence GBS tags could be genetically mapped in maize, the binomial test of segregation was repeated versus this high density framework map, with a threshold of *p*<0.0001.

Software for the sequence filtering and the mapping analysis was written in Java and is available on SourceForge (http://sourceforge.net/projects/tassel/). This software is part of the TASSEL package but is not currently implemented in the TASSEL GUI.

## Results

### Read quantity and quality

Because we are interested in enabling genome wide association studies (GWAS) in maize, a species where linkage disequilibrium decays within two to three kbp [30], we need to identify markers that cover around one million genomic locations. For this reason we chose to use *Ape*KI, a RE that should cut frequently in the maize genome because it recognizes a degenerate five bp DNA sequence. Of course, if less genome coverage is desired, the protocol can be easily modified to use enzymes that recognize six or more bp.

Out of 1,146,449 high-quality (filtered) reads from IBM parental line B73, 1,125,731 (98%) could be aligned with the maize genomic DNA sequence. BLAST results indicated that the majority of non-aligning reads represented maize sequences that were absent in the reference genome version used for the analysis (B73 RefGen v1). Of the 868,336 GBS sequence reads that aligned perfectly to the maize genome (no mismatches), 673,354 (78%) mapped to single genomic locations while 194,982 (22%) mapped to multiple locations, 87,271 (10%) aligned to <5 sites while 107,711 (12%) mapped to ≥5 sites).

Sequences from the maize IBM mapping population (276 RILs) were collected in six lanes of a single flow cell at 48-plex. On average, 2,090 Mbp of DNA sequence data were collected per lane. From a total of 145,836,644 raw reads, 102,505,713 (70%) were “high quality reads” that passed the Illumina filter while 120,438,739 (83%) contained the barcode and the cut site and no “Ns” within the first 72 bases and were not adapter/adapter dimers. This observation indicates that, overall, the Illumina filtering parameters seem to be underestimating read quality. Hence, to maximize the amount of useful data, we worked with raw reads from the qseq file. Very few adapter dimers were detected (78,375 or <0. 06% of the raw reads). Out of the 25,397,905 rejected raw reads (17% of total reads), only 1,096,513 (0.75% of the total reads) were discarded solely because of “Ns” in the first 72 bases. The remainder of rejected reads was comprised of adapter/adapter dimers and sequences that did not contain the barcode and cut site (24,223,017 reads). Of the 1,096,513 reads discarded solely because of “Ns”, only 36,009 contained a single “N” and only 21,005 contained two “Ns”, whereas the majority (1,039,499) contained more than two “Ns”.

**Figure 3:**
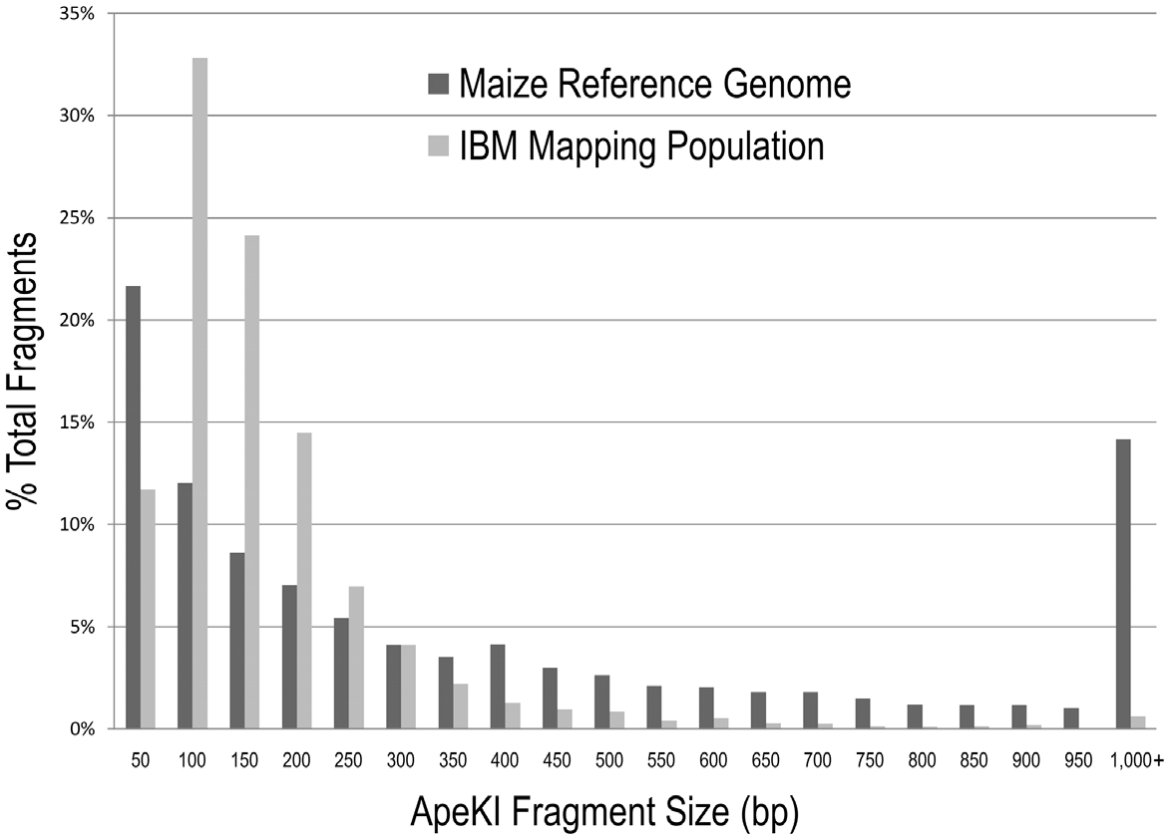
Fragment size distributions of a virtual *Ape*KI digest of the maize genome and unique (single-copy) *Ape*KI sequence tags from the maize IBM mapping population. Note that for size bins on the x-axis “50” denotes a bin of size 1–50 bp, “100” denotes a bin of size 51–100 bp, etc. The reference genome employed for the maize virtual digest was B73 RefGen v1.

From six sequencing lanes, we identified 809,651 sequence tags (at least five times) from one or both flanks of 654,998 of the 2.1 million *Ape*KI cut sites lying within the single copy genomic fraction. These 0.81 million 64 bp sequence tags cover 51.8 Mbp, or 2.3% of the maize genome. We also observed that the *Ape*KI libraries showed a preponderance of smaller fragments (Figure 3), resulting from both a bias toward production of small fragments during the PCR step of library construction, and precise spatial requirements for optimal cluster formation on the sequencing flow cell (i.e., longer fragments produce diffuse clusters that result in low sequence signal intensity). Fragments under 64 base pairs result in the presence of either the common adapter or an internal *Ape*KI recognition sequence (from partial digestion or chimera formation) within 64 bases of the end of the barcode. These were fairly common; out of the 120,438,739 reads that passed our initial filtering criteria (possessing a bar code and cut site, etc), 20,585,840 (17%) were from fragments less than 64 bases in length. As noted in the Methods, these were truncated accordingly and filled to 64 bases with polyA.

### Barcode optimization

Our preliminary studies using RE-digested DNA samples and a small number of same-length (8 bp) barcodes showed a substantial decline in read quality in multiplexed sequencing reactions compared to control DNA or other barcoded DNA samples that did not include restriction sites (data not shown). This finding suggests that presence of the invariant restriction site recognition sequence at the beginning of each read (i.e., low 5′ sequence variation) caused base calling errors in subsequent cycles, probably because proper sequence phasing on the Illumina Genetic Analyzer is dependent on detecting 12 random nucleotides at the beginning of each sequence. The presence of the invariant RE cut-site at bases nine to 12, therefore, violates the phasing model assumptions (http://www.illumina.com/Documents/products/technotes/technote_rta_theory_operations.pdf). Incorporation of variable length barcodes substantially improved base calling accuracy, although it still appears that the Illumina algorithm sometimes underestimates read quality. Reads that did not pass the Illumina filter sometimes perfectly matched a 64 base tag that was segregating in our mapping populations.

### Sample representation

**Figure 4:**
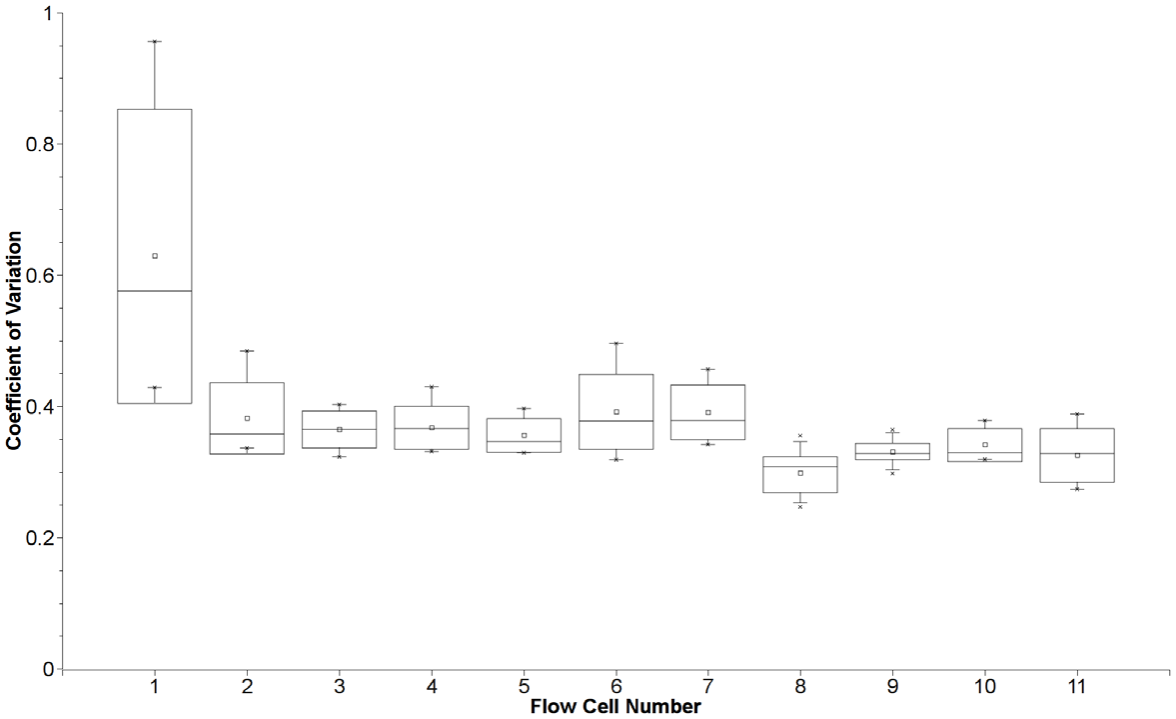
Coefficient of variation of GBS reads per sequencing channel for sequential sequencing runs. Each flow cell comprised 6 or 7 sequencing channels. Large boxes represent the standard deviation of the number of reads per sample; whiskers denote minimum and maximum values; small squares are the median values; and lines extending across the boxes are the means for each run. Flow cells are ordered sequentially by run date; number 1 is the first sequencing run and number 11 denotes the last run. The GBS read data from the maize IBM population is contained in flow cell 1. The large variation in reads per sample from this flowcell was due to inconsistent pipetting during robotic liquid handling. Subsequent adjustments to our robotic protocols improved evenness among samples (see flowcells 2–11).

The six lanes of the maize IBM population sequencing run yielded 120,438,739 GBS reads that contained the barcode and the *Ape*KI cut-site (or 20,073,123 reads per lane). On average, 436,372 reads were produced per DNA sample and 95% of samples generated at least 125,000 reads. Evenness of sample representation among the maize IBM RILs was acceptable but not optimal. In our best lane from the IBM flow cell, the coefficient of variation (cv = standard deviation/mean) for the number of reads containing the appropriate barcode and the cut site was roughly 43% among samples and, among the six lanes, 39.8% of the variance was attributed to DNA sample. Subsequent adjustments to our robotic liquid handling protocols, however, have resulted in greater evenness among samples (Figure 4). Regardless of the disproportionate sample representation, we were still able to map a minimum of 90,000 sequence tags in the poorest performing IBM samples.

**Figure 5:**
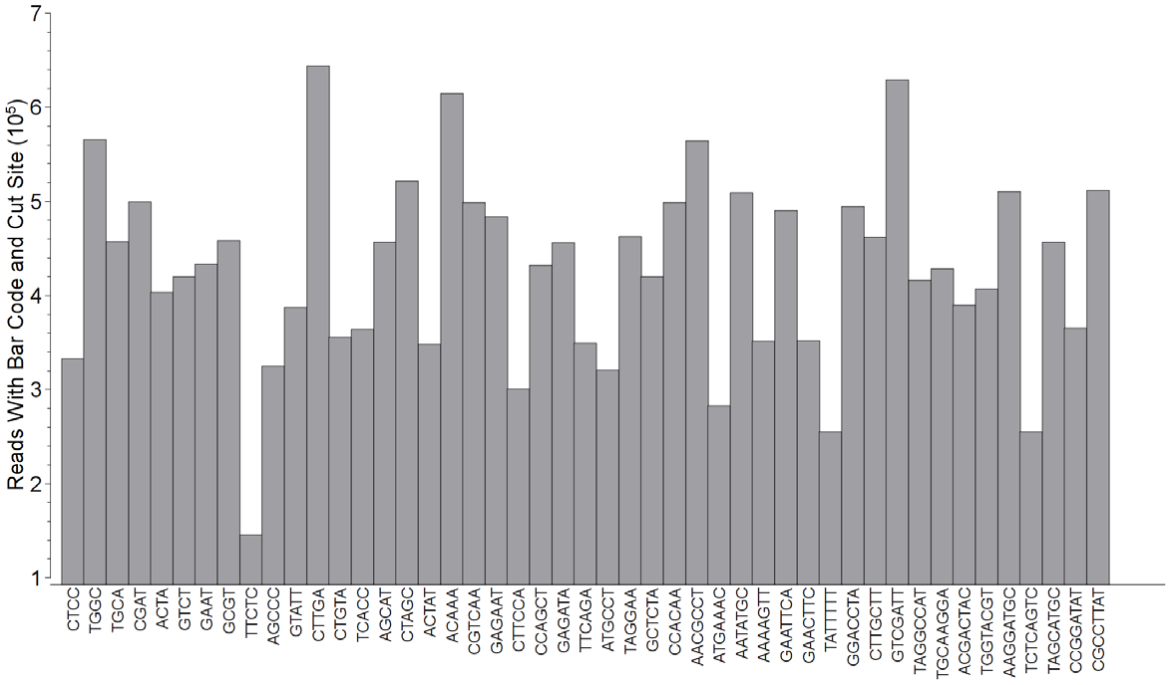
Distribution of reads across 43 barcoded samples in a single flow cell lane for the Oregon Wolfe Barley population.

Preliminary results for barley were slightly better with respect to uniform sample representation (Figure 5). The one channel of the sequencing run produced 27.5 million reads. On average, 427,130 reads were produced per DNA sample (minimum = 145,648; maximum = 643,631) with a coefficient of variation (cv) of 23% (Figure 5).

### Mapping and SNP validation

Analysis of the maize IBM population provided a preliminary evaluation of the genetic value of multiplex GBS skimming. Overall 25,185 biallelic 64 base tags were genetically mapped to their physically closest anchor SNP. No corresponding alternate allele was found for an additional 584,119 tags. By treating these as dominant data (i.e., either present or absent in each RIL), 167,494 could be placed upon the framework map of 25,185 biallelic sequence tags based upon segregation. Alignment to the reference genome detected unique physical positions for 133,129 of the dominant markers, 90.8% of which agreed with the genetic positions.

**Figure 6:**
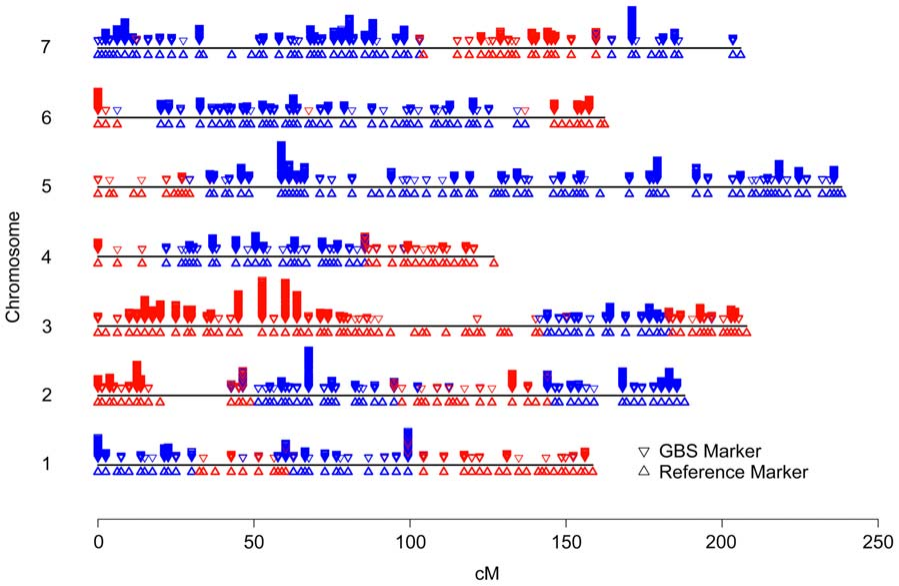
Barley GBS marker validation using a single DH line (OWB003). Upright triangles denote positions of markers on the reference genetic map and downward triangles indicate GBS reads mapped in this study. Multiple sequence reads are stacked and colors indicate chromosomal segments in OWBOO3 originating from dominant (blue) or recessive (red) parental lines.

After filtering for tags present in at least 20% of the lines, 2.1 million unique barley tags were retained. These tags were mapped to the OWB framework map of 2,382 markers [31] by considering tags as dominant markers and anchoring the tags using the reference map. Prior to mapping, the genetic map was collapsed to retain only markers that contained unique linkage information in the subset of 43 lines (i.e., SNPs at the same map position were removed) leaving 436 biallelic markers. **I**n all, we mapped 24,186 sequence tags onto the barley genetic map. To determine the utility of using the sequence tags as genetic markers, cross validation was conducted for one of the OWB lines (OWB003). Tags were mapped without OWB003 and coded according to whether they were present in either the dominant or recessive OWB parent. A graphical genotype of the excluded, control line, OWB003, showed almost perfect agreement between the reference markers and GBS regarding chromosome segment parent of origin (Figure 6). A cross validation error was scored if a previously mapped SNP and a GBS tag disagreed on parent of origin. GBS markers occurring near the OWB003 recombination break points cannot be unambiguously assigned and were excluded when determining genotyping accuracy. Of the 4,596 mapped GBS reads present in OWBOO3, 4,533 (99%) identified the correct parent of origin.

## Discussion

### GBS offers an alternative to complex, expensive protocols

The value of reducing genome complexity with REs coupled with multiplex NGS for high-density SNP discovery and genotyping was originally demonstrated with restriction site-associated DNA (RAD) tags [20]. In the RAD protocol, genomic DNA is digested with a six to eight base-cutter RE and a barcoded adapter is ligated to compatible sticky ends. For multiplex sequencing, DNA samples, each with a different barcode, are pooled, randomly sheared, size selected (300–700 bp), and a second adapter is ligated after polishing and filling ends. The RAD library preparation procedure is substantially more complicated than the one presented here. In addition to its simplicity (no fragment size selection and few enzymatic and purification steps), our protocol is time and cost efficient through its use of a single well for genomic DNA digestion and adapter ligation. These processes can be done in the same buffers so that no additional transfers are needed. Currently, the favored enzymes (*Ape*KI and T4 ligase) do not have complementary temperature regimes so simultaneous digestion and ligation is not possible unless we substitute an expensive, thermostable ligase.

Recently, a multiplex NGS protocol appropriate for *Drosophila* and other small-bodied species has been published [32]. Although this protocol is similar to the one reported here, it still includes a fragment size selection step and variation in the number of reads between samples was considerably higher (cv = 89%) [32] than what we observed (cv = 23–43%). Clearly for any multiplex sequencing protocol, accurate quantification of high molecular weight DNA remains a procedural bottleneck, and is the most likely source of sample-to-sample variation in sequence coverage. DNA quantification using intercalating dyes and spectrophotometry give correlated but not very consistent results. Developing a more precise, cheap, high-throughput DNA quantification protocol, therefore, remains an area where this method can be improved. The performance of the barcodes, themselves, is less problematic because, over time, poor performing barcodes can be identified and removed from the protocol.

### GBS does not use 5′-phosphorylated adapters

Unlike other protocols, GBS employs unmodified adapters (i.e., without 5′-phosphate groups). As a result, only one adapter strand is covalently bound to the ends of restriction fragments. As long as DNA samples are not denatured prior to the pooling and PCR steps, however, *Taq* polymerase rapidly fills the 3′ recessed ends in the presence of dNTPs. End filling occurs either by immediate displacement of the non-ligated adapter strands at low temperatures (during the assembly of PCR reactions) or following the early dissociation of short, non-ligated strands during the initial heating step of the PCR [33]. Use of unphosphorylated adapters has the added benefits of destabilizing formation of adapter dimers during library preparation and reducing reagent costs.

### GBS does not employ divergent “Y” adapters

Standard libraries for Illumina sequencing are prepared by ligating a single “Y” or “forked” adapter to both ends of genomic DNA fragments [26]. These adapters, made by annealing oligonucleotides with both complementary and non-complementary sequences, have, at one end, a region of double stranded DNA that is required for T4 DNA ligase to join adapters to genomic DNA. The other end of the adapter is comprised of single stranded, divergent sequences that serve as binding sites for a pair of primers that, after PCR, generate DNA fragments that have different adapter sequences on each end.

The GBS protocol employs two different double stranded adapters (barcode and common) that are ligated simultaneously to restriction fragments with “sticky” ends. This means that any combination of adapters (barcode/common, barcode/barcode, or common/common) may be joined to genomic DNA fragments. Because same-ended DNA strands bind to the flowcell but do not produce DNA sequence on the Illumina platform (those having only barcode adapter sequences are cleaved from the surface prior to reverse-terminator sequencing and those with two common adapter ends lack a binding site for the PE1 sequencing primer), one might predict a reduction in the number of reads per lane with the GBS protocol. Read numbers, however, have equaled or exceeded the specifications of the DNA sequencing instruments (both the Illumina GAII and, more recently, the Illumina Hi-Seq). The lack of impact on read number is most likely because same-ended fragments occupy little physical space on the flowcell. This reasoning seems counterintuitive in light of the fact that half the fragments applied to the flowcell are predicted to have the same adapter ends. Because same-ended strands are able to utilize only one of the two oligonucleotides that coat the flowcell surface during bridge amplification [34], however, formation of DNA clusters (colonies) is inefficient. As a result, these slow-growing, sparse clusters are rapidly overrun by “normal” colonies derived from DNA strands with different ends that prime off both oligonucleotides.

### GBS accesses regulatory regions and sequence tag mapping requires no reference genome

As more information is gathered, it is becoming apparent that regulatory regions controlling the expression of plant genes responsible for agronomically important phenotypes are often located in non-coding DNA. For example, regulatory regions of maize genes *vgt1* [35], *tb1* [36] and *b1* [37] are located 60 to 150 kb from the structural gene. Therefore, systematic discovery and mapping of genetic diversity should not be limited to coding regions. In this sense, the GBS procedure allows access to any sequence within “low-copy” genomic regions, including transposable elements and repeat regions that have not proliferated extensively.

Another advantage to the GBS approach is that a reference genome need only be developed neighboring the restriction sites, and this can be done in the process of sample genotyping. In such cases, the consensus of the read clusters across the sequence tagged sites becomes the reference. Alternatively, for kinship analyses and genomic selection in the absence of a reference genome, the tags can simply be treated as dominant markers. While not addressed here, there has also been tremendous progress in the imputation of missing data. In biparental mapping populations of species with a reference genome, this can be done with extremely high accuracy [5], and even in more diverse material, imputation accuracies over 99% permit low coverage. In the case of maize, we envision performing whole genome sequencing on a few thousand lines and then projecting their polymorphisms onto hundreds of thousands of additional lines via GBS and imputation.

We have shown that for an expenditure of $8,000 (USD), approximately 200,000 maize markers can be identified and mapped in a very short time. With the methods outlined here, we can process 336–672 samples (48 or 96 samples per channel ×7 channels per flow cell) simultaneously. Multiplexes up to 384 per lane (2,688 samples per sequencing run) are becoming possible as read density improves on sequencers. The economy of scale associated with these improvements is rapidly pushing genotyping below $20 per sample. Projected gains in the near future could result in a further four to five fold reduction to $5 or less per sample. Soon, plant breeders may conduct genomic selection on a novel germplasm or species without first having to develop any prior molecular tools, or conservation biologists may determine population structure without prior knowledge of the genome or diversity in the species. These exciting new avenues for applying GBS to breeding, conservation, and global species and population surveys are now poised to become an indispensable component of future biology.

## Supporting Information

Text S1 **Optimizing GBS for Other Species.**
(DOCX)

Figure S1 **Experion® output showing fragment size distribution of an “unoptimized” maize GBS library.** Note that the x-axis denotes seconds (elution time) and not fragment size (bp). Two discrete peaks are observed, the primer dimer peak at around 45 seconds (∼70 bp), the adapter dimer peak between 51 and 52 seconds (∼128 bp). These are followed by a broad peak, the GBS library, occurring between 55 and 65 seconds.
(TIFF)

Figure S2 **Experion® output showing fragment size distribution of a GBS library after adapter amounts were optimized.** Note that the adapter dimer peak has disappeared.
(TIFF)

Table S1 **GBS barcode sequences for** ***Ape*** **KI adapters.**
(DOCX)
