# Supplementary material for: A Robust, Simple Genotyping-by-Sequencing (GBS) Approach for High Diversity Species
Source: PLoS One. 2011 May 4;6(5):e19379. doi: 10.1371/journal.pone.0019379 (PMC3087801; doi:10.1371/journal.pone.0019379)
Supplement: Text S1 — Optimizing GBS for Other Species. (DOCX) [file pone.0019379.s001.docx]

**Supporting Information**

**Optimizing GBS for Other Species**

**Identification and testing of potentially suitable restriction enzymes (REs)**

Select restriction enzymes (REs) that are methylation-sensitive (do not cut frequently in the major repetitive fraction of the genome) and produce overhangs (“sticky ends”) within the RE recognition site (do not use “off-set” cutters). Genome size of the species of interest and the amount of sequence overlap desired among samples will influence whether frequent- (REs with four- or five-bp recognition sequences) or infrequent- (six or more bp recognition sequence) cutters should be used. Good sequence overlap among samples and a fair sized pool of “smallish” fragments are attained using REs with six base recognition sequences (eg., *Pst*I).

To test whether DNA extractions are of sufficient quality for library construction, run an agarose gel with aliquotes of cut and uncut sample DNA. Because methylation-sensitive enzymes cleave only at unmethylated recognition sites, only a small portion of cut-sites may be digested, depending on the amount of repetitive DNA present in the genome of interest. Therefore, the digestion test should be done with a cheap RE that is not methylation-sensitive (e.g., *Hin*dIII or *Eco*RI).

**Optimizing reaction conditions**

If the RE cuts fairly well at room temperature (25^◦^C) and remains active for extended digestion times, it may be possible to combine digestion and adapter ligation steps. In this case, we suggest performing the digestion/ligations in the RE buffer spiked with 1mM ATP. Incubate overnight at room temperature. Initially, use the adapter amounts recommended in the GBS protocol (see text) and evaluate library fragment size distribution on a BioRad Experion®, Bioanalyzer® (Agilent) or similar instrument. If adapter dimers (peak at 128bp) represent > 0.5% of the total number of fragments contained within the library, the amount of adapters appropriate for your species or DNA extractions should be determined empirically (i.e., by titration). **Caution:** if you do not use T4 ligase from New England Biolabs, activity is usually expressed in Weiss Units (1 Weiss Unit = 67 NEB Cohesive End Ligation Units).

**Adapter titration**

The proper ratio of adapters to sample DNA “sticky” ends is critical to the success of this method. A scarcity of adapters encourages chimera formation among sample DNA molecules while an overabundance results in formation of adapter dimers. Moreover, adapter dimers will produce DNA sequence data, thus wasting reagents and machine time. The REs used in GBS are methylation-sensitive. The number of cut-sites, therefore, will depend on the amount and distribution of highly methylated repetitive DNA within the genome of interest. Because it is difficult to estimate the number of restriction sites produced by these REs, adapters are titrated against a set amount of digested DNA to empirically determine the correct ratio of adapters to sample DNA ends. The titration needs to be performed only once for a given species or DNA extraction. The following protocol uses eight different adapter concentrations. If there is little variation in genomic DNA sample quality (samples must be high molecular weight and free of RNA and other contaminants) and concentrations are fairly even across the population of samples to be sequenced, chose a representative sample DNA for the titration experiment. If sample quality is extremely variable, re-extract problematic DNAs before making libraries.

**Digest DNA**

In an 8 strip tube set up each of the 8 reactions as follows:

| **Reagent** | **Volume (**µL) |
| --- | --- |
| Genomic DNA (100ng / µL) | 2 |
| NEB Buffer 3 (10X) | 2 |
| *Ape*KI (4U/µL) | 1 |
| dH_2_0 | 15 |
| **Total** | **20** |

Tap to mix.

- Spin briefly in tabletop centrifuge.
- Incubate at 75°C for 2 hours.

# Ligate varying amounts of adapters

| **Reagent** | **Tube 1 (µL)** | **Tube 2 (µL)** | **Tube 3 (µL)** | **Tube 4 (µL)** | **Tube 5 (µL)** | **Tube 6 (µL)** | **Tube 7 (µL)** | **Tube 8 (µL)** |
| --- | --- | --- | --- | --- | --- | --- | --- | --- |
| Digested DNA | 20 | 20 | 20 | 20 | 20 | 20 | 20 | 20 |
| NEB Ligase buffer (10X) | 5 | 5 | 5 | 5 | 5 | 5 | 5 | 5 |
| Adapter mix* (0.3ng/ µL each adapter) | 6 | 8 | 12 | 14 | 16 | 18 | 20 | 24 |
| dH_2_O | 18 | 16 | 12 | 10 | 8 | 6 | 4 | 0 |
| NEB T4 DNA ligase (400 CELU*****/ µL) | 1 | 1 | 1 | 1 | 1 | 1 | 1 | 1 |
| **Total** | 50 | 50 | 50 | 50 | 50 | 50 | 50 | 50 |

*1 CELU = 0.015 Weiss Units

**Note:** Use only one set of adapter pairs (i.e., one of the 96 barcode adapters with a barcode listed in Table S1 along with the common adapter).

- Add the ligase to the side of the tube so that it is added to all the reaction tubes at the same time.
- Spin down briefly in tabletop centrifuge.
- Incubate at 22°C for 60 minutes.
- Deactivate the ligase by incubating at 65°C for 30 minutes. Cool to 4°C.

# Clean-up

- Cleanup with Qiagen PCR cleanup kit per kit instructions.
- Elute in 50µl elution buffer (EB).

# PCR Amplification

For each of the 8 reactions from above, set up a PCR reaction as follows:

| **Reagent** | **Volume (µL)** |
| --- | --- |
| DNA from previous step | 10 |
| NEB 2x Taq Master Mix | 25 |
| PCR Primer Mix (12.5 pmol/µL each primer) | 2 |
| dH_2_O | 13 |
| **Total** | **50** |

- Amplify using the following PCR cycling protocol:

a. 5 minutes at 72°C

b. 30 seconds at 98°C

c. 18 cycles of:

10 seconds at 98°C

30 seconds at 65°C

30 seconds at 72°C

d. 5 minutes at 72°C

e. Hold at 4°C

## Clean-up

- For each of the PCR reactions, purify with Qiagen PCR cleanup kit per kit instructions.
- Elute in 30µL Qiagen EB.

## Evaluate libraries

Figure S1 shows output from the BioRad Experion® for a maize GBS library made with an excess of adapters. Note that a large adapter dimer peak is evident around 128 bp. The very small peak around 70 bp consists of PCR primer dimers.

Run each of the 8 samples on the Experion® according to manufacturer’s instructions. Determine the amount of adapter that produces a good library but shows **no adapter dimer peak** (Figure S2). Use **half** the amount of adapter derived in this experiment when making multiplex sequencing libraries (200ng of digested DNA is used in the adapter titration experiment but only 100ng is needed for GBS library construction).

**Mapping reads**

Genetic maps, mapping populations and detailed pedigrees exist for many species without sequenced genomes. If samples from large mapping populations (a few hundred individuals) are available, linkage relationships can be established for sequence polymorphisms (biallelic or dominant markers) discovered by GBS. Here, the consensus of the read clusters across the sequence tags becomes the reference. If mapping populations are not available, SNPs detected by GBS can be converted to sequence-based markers and scored on a variety of different platforms. If nothing is known about sequence variation in the species of interest, we suggest beginning with a “pilot” study using a limited number of samples that, based on morphology, biological traits or geographical distribution, are expected to show the maximum amount of diversity.
