# Supplementary figures and images for: A Robust, Simple Genotyping-by-Sequencing (GBS) Approach for High Diversity Species
Source: PLoS One. 2011 May 4;6(5):e19379. doi: 10.1371/journal.pone.0019379 (PMC3087801; doi:10.1371/journal.pone.0019379)

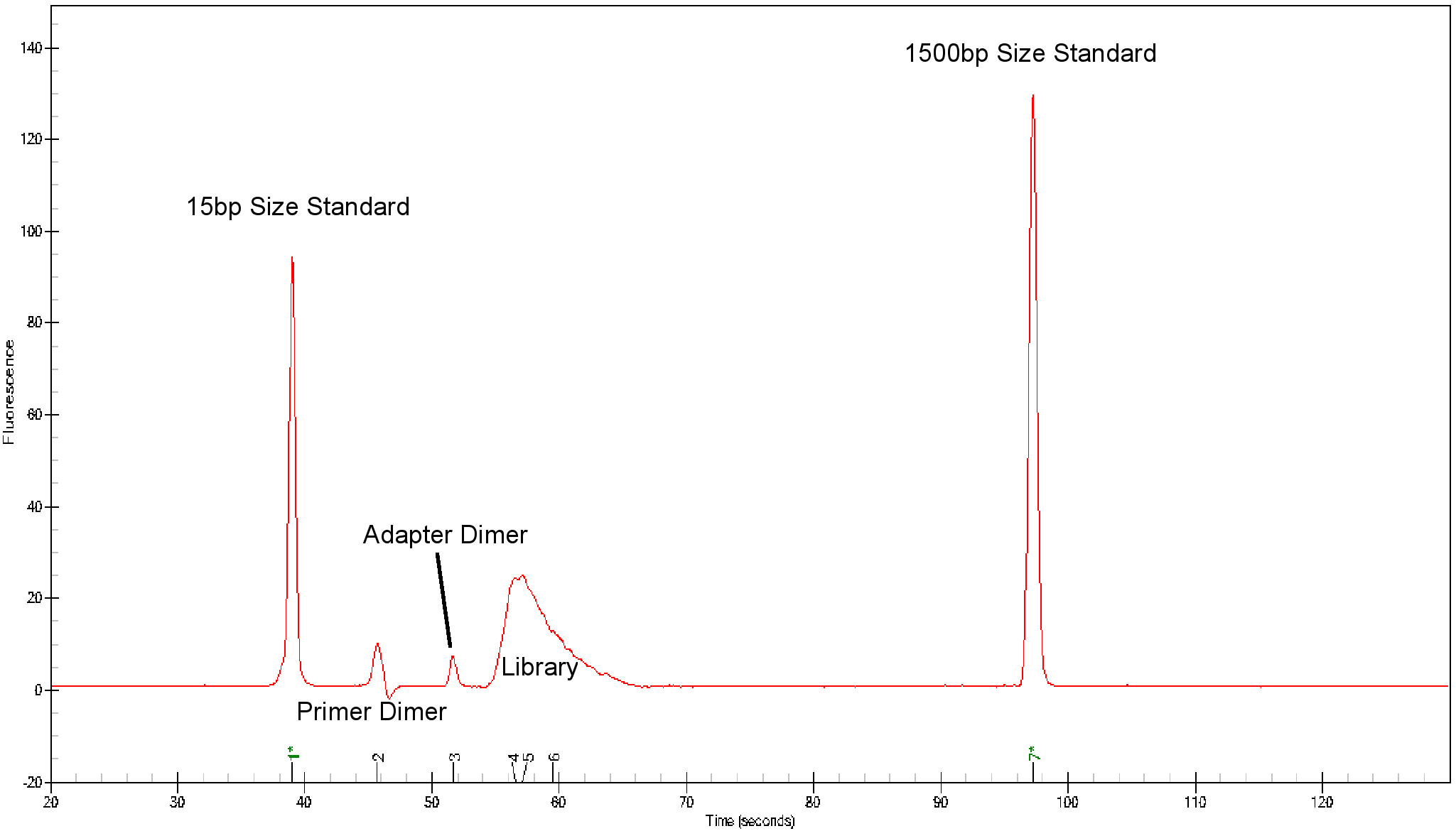

Supplement: Figure S1 — Experion® output showing fragment size distribution of an “unoptimized” maize GBS library. Note that the x-axis denotes seconds (elution time) and not fragment size (bp). Two discrete peaks are observed, the primer dimer peak at around 45 seconds (∼70 bp), the adapter dimer peak between 51 and 52 seconds (∼128 bp). These are followed by a broad peak, the GBS library, occurring between 55 and 65 seconds. (TIFF) [file pone.0019379.s002.tif]

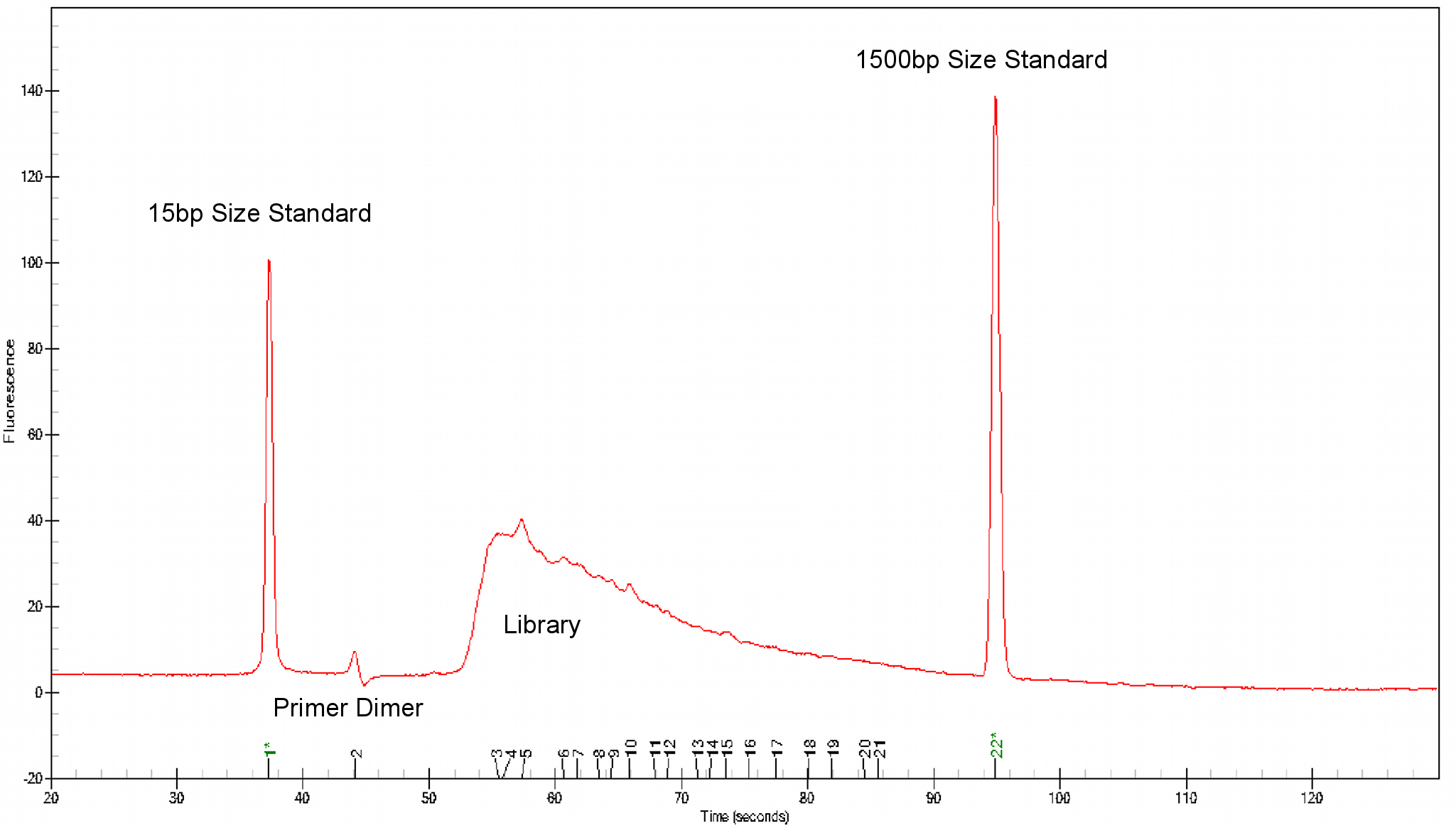

Supplement: Figure S2 — Experion® output showing fragment size distribution of a GBS library after adapter amounts were optimized. Note that the adapter dimer peak has disappeared. (TIFF) [file pone.0019379.s003.tif]
