# Supplementary material for: A Robust, Simple Genotyping-by-Sequencing (GBS) Approach for High Diversity Species
Source: PLoS One. 2011 May 4;6(5):e19379. doi: 10.1371/journal.pone.0019379 (PMC3087801; doi:10.1371/journal.pone.0019379)
Supplement: Table S1 — GBS barcode sequences for Ape KI adapters. (DOCX) [file pone.0019379.s004.docx]

| CTCC | TTCTC | TCGTT | CTATTA | AATATGC | TGCAAGGA |
| --- | --- | --- | --- | --- | --- |
| TGCA | AGCCC | GGTTGT | GCCAGT | ACGTGTT | TGGTACGT |
| ACTA | GTATT | CCAGCT | GGAAGA | ATTAATT | TCTCAGTC |
| CAGA | CTGTA | TTCAGA | GTACTT | ATTGGAT | CCGGATAT |
| AACT | ACCGT | TAGGAA | GTTGAA | CATAAGT | CGCCTTAT |
| GCGT | GCTTA | GCTCTA | TAACGA | CGCTGAT | AACCGAGA |
| CGAT | GGTGT | CCACAA | TGGCTA | CGGTAGA | ACAGGGAA |
| GTAA | AGGAT | CTTCCA | TATTTTT | CTACGGA | ACGTGGTA |
| AGGC | ATTGA | GAGATA | CTTGCTT | GCGGAAT | CCATGGGT |
| GATC | CATCT | ATGCCT | ATGAAAC | TAGCGGA | CGCGGAGA |
| TCAC | CCTAC | AGTGGA | AAAAGTT | TCGAAGA | CGTGTGGT |
| TGCGA | GAGGA | ACCTAA | GAATTCA | TCTGTGA | GCTGTGGA |
| CGCTT | GGAAC | ATATGT | GAACTTC | TGCTGGA | GGATTGGT |
| TCACC | GTCAA | ATCGTA | GGACCTA | ACGACTAC | GTGAGGGT |
| CTAGC | TAATA | CATCGT | GTCGATT | TAGCATGC | TATCGGGA |
| ACAAA | TACAT | CGCGGT | AACGCCT | TAGGCCAT | TTCCTGGA |
